# Perception of donor breast milk and determinants of its acceptability among mothers in a developing community: a cross-sectional multi-center study in south-east Nigeria

**DOI:** 10.1186/s13006-018-0189-2

**Published:** 2018-11-14

**Authors:** Kenechukwu K. Iloh, Chidiebere DI. Osuorah, Ikenna K. Ndu, Isaac N. Asinobi, Ijeoma N. Obumneme-Anyim, Chijioke E. Ezeudu, Ukoha M. Oluchi, Onyinye U. Anyanwu, Uchenna Ekwochi, Christian C. Ogoke, Adaeze C. Ayuk, Herbert U. Obu

**Affiliations:** 1 University of Nigeria Teaching Hospital|, Nsukka, Nigeria; 2 Child Survival Unit, Medical Research Council UK, The Gambia Unit, Fajara, Gambia; 3 Enugu State University of Science and Technology, Enugu, Enugu state Nigeria; 4 Nnamdi Azikiwe University, Nnewi, Anambra state Nigeria; 5 Federal Medical Center Owerri, Owerri, Imo state Nigeria; 6 Federal Teaching Hospital Abakiliki, Abakaliki, Ebonyi state Nigeria; 7 Federal Medical Center Umuahia, Umuahia, Abia state Nigeria

## Abstract

**Background:**

Due to the health and economic benefits of breast milk, the World Health Organization (WHO) recommends that for infants who cannot receive breast milk from their own mothers, the next preferred option is donated breast milk. This recommendation is however rarely practiced in most developing countries where donor milk is not widely accepted.

**Methods:**

This cross-sectional multi-center study enrolled mothers attending antenatal or pediatric clinics in six tertiary institution in south-east Nigeria using purposive and convenient sampling method. Data collection was done using pretested questionnaires. The study aimed to assess the knowledge, acceptability and willingness to donate breast milk and/or use donated breast milk for their infants It also explored factors that determine this behavior.

**Results:**

A total of 1235 mothers participated; 39% (480/1225) have heard about the concept of donor milk, while only 10% (79/759) and 7% (81/1179), respectively, had adequate knowledge of the concept and policy on donor milk. Sixty percent indicated willingness to use donor milk or donate breast milk if need arises. Respondents with lower age (*p* = 0.049) and with higher occupational status (*p* = 0.001) were more likely to have adequate knowledge of donor breast milk, while respondents with lower educational attainment (*p* = 0.002) and those who are non-Christians (*p* = 0.004) were more likely to request financial inducement for donating their breast milk. Adequate knowledge of the concept of donor milk (*p* = 0.001), preference of donor milk to infant formula (*p* = 0.001) and requirement of financial remuneration (*p* = 0.001) were the only significant predictors of willingness to donate and/or receive donated breast milk.

**Conclusion:**

The knowledge of the concept of donor breast milk and awareness of policies regulating its practice in Nigeria is low, but the prospect of its acceptability is high among mothers surveyed in south-east Nigeria. Targeted public education by relevant government agencies in collaboration with clinicians, community and religious leaders about the concept of donor breast milk to families may help increase the acceptance and practice of donating breast milk and/or use of donated breast milk among mothers in the region.

## Background

Breastmilk has been well-established as the most optimal source of nutrient for infants particularly in the first six months of life [1]. This is partly because it contains bioactive substances that are essential to the development of the newborn’s immature immune system. This singular attribute of breastmilk makes it even more important for the preterm and/or low birthweight newborn whose weak immune system is vital for its survival. Apart from the immune system, other long-term benefits of the use of human breast milk in preterm is well documented. These include but not limited to decreased incidences of retinopathy and necrotizing enterocolitis of the newborn, better neuro-cognitive development and lower risk of development of childhood obesity and diabetes [2–4]. It has been noted that newborns most susceptible to morbidity and mortality are those at greatest risk of not receiving the lifesaving benefits of breast milk [5].

Based on the benefits, the World Health Organization (WHO) recommends that all mothers should feed their infants solely with breast milk, exclusive of any other nutritional sources for the first six months of their infant’s life [6]. The recommendation further states that for infants who cannot receive breast milk from their own mothers, the next preferred option is donated human breastmilk. Donated breastmilk according to the National Institute of Health and Clinical Excellence (NICE) is breast milk expressed by a mother that is then processed by a donor milk bank for use by a recipient that is not the mother’s own baby [7]. The use of donor breastmilk over infant formulas in vulnerable newborns has been shown to significantly impact on neonatal mortality rates in countries around the world. In Brazil for instance, there has been a drastic reduction in neonatal mortality by a whopping 73% between 1990 and 2013, since it incorporated milk banks into its newborn health policy [8, 9]. Unfortunately, most developing countries with the highest burden of global neonatal mortality lag in the implementation of donor breastmilk as the best alternative for vulnerable infants.

In Nigeria, it is estimated that of the seven million children born every year only about 25% are exclusively breastfed from 0 to 6 months of age [10]. Sadly, this poor practice of breastfeeding has to a certain degree been implicated in the current high rate of neonatal morbidity and mortality in the country [10]. Even more worrying is the fact that there is currently no human breastmilk bank in the whole of the west Africa sub-region. Due to strong religious and cultural belief system, establishing human breastmilk bank in these settings for use in precarious situation where maternal milk is limited or not available such as prematurity, low birthweight or orphaned newborns requires an in-depth consideration of the acceptability of the concept by mothers in these settings. There is little discussion in local medical literature regarding the knowledge and willingness to use donor breastmilk among mothers in many developing communities. This study therefore sets out to assess the perception of the concept of donor breastmilk among mothers in several communities in south-east Nigeria. It further evaluated the factors that determine the willingness to participate in donation and/or use of donor breast milk among these mothers.

## Methods

This cross-sectional multi-center study was conducted concurrently over a six months period between August 2016 to January 2017 in six different tertiary health facilities namely the University of Nigeria Teaching Hospital (UNTH), Enugu, Enugu State University Teaching Hospital (ESUTH), Enugu, Federal Teaching Hospital Abakiliki (FETHA) Ebonyi State, Federal Medical Centre Owerri, Imo State, Federal Medical Centre Umuahia, Abia State and Nnamdi Azikiwe University Teaching Hospital (NAUTH) Anambra State. Each of these hospital offers specialized medical services and serves as a referral center to primary, secondary and private health facilities from within and outside their respective state. These six tertiary health institutions are all located in the south-east area of Nigeria and were chosen out of the 10 tertiary institutions in the region using convenient sampling method. This was based on the presence of one or more authors of this work being a staff in the institutions and/or supervisor(s) of the data collection team. The study aimed to enroll two hundred and fifty mothers attending antenatal and postnatal clinics in each of the selected center using purposive sampling method. A structured pre-tested questionnaire was administered by self and/or interviewer-based supervision depending on the educational abilities of respondent.

### Inclusion and exclusion criteria

All women who were pregnant or breastfeeding or have a child that is less than one year attending the antenatal and/or pediatrics clinic in the various study centers were included in the study while mother with children one year or above and those who refused to give consent to participate were excluded from the study.

### Sample size determination

The sample size was calculated using a single population proportion formula with 95% confidence level, exclusive breastfeeding rate of 25% in Nigeria [10] and a non-response rate of 10%. This gave a minimum sample size of 317. To enhance the power of this study, we aimed to enroll 250 mothers from each of the 6-study site giving a working total sample size of 1500 respondents.

### Measures

The study variables were collected into the relevant sections of the questionnaire. In the first section *predictor variables* which included sociodemographic features of participants were collected and categorized as follows: i*)* Respondents age in years categorized as, < 20, 20–29, 30–39 and ≥ 40 years. ii*)* Educational level of respondents categorized as primary education or less (≤ 6 years of education), completed secondary (6–12 years of education) and post-secondary education (> 12 years of education). iii*)* Respondents occupation was categorized as unemployed (i.e. house wives, students etc.), unskilled (i.e. petty traders, cleaners, hairdressers etc.), semi-skilled (clerical secretaries, teachers etc.) and skilled (doctors, bankers, accountants etc.). iv*)* Socioeconomic class of respondent was calculated based on maternal education and paternal occupation using a scale [11] developed and validated for Nigeria and other developing setting and categorized as low, middle and high. v*)* Religion of respondent was grouped into Christianity, Islam, Traditional and others. This was further re-grouped into Christians and non-Christians. vi*)* Respondents tribe was classified into Igbo, Hausa, Yoruba and others for minorities tribes. vii*)* Marital status was categorized as married and single. viii*)* Spouse age was categorized as < 30 years, 30–39 years, 40–49 years and ≥ 50 years. ix*)* Spouse education was primary education or less, completed secondary and post-secondary education.

The second section of the questionnaire collected information on *outcome variable* that measured parameters that assessed respondents’ attitude towards donor breast milk. Donor breast milk was defined based on the NICE clinical guideline [7]. These parameters included i*)* If respondents have ever heard of the concept of breast milk donation (categorized as yes or no). ii*)* Knowledge of the concept of donor milk (categorized as none, partial and adequate). iii*)*. Awareness of policy on breast milk donation was categorized as yes or no based on the knowledge of section 2.2.4 of the policy that states that for motherless and/or adopted infants’ relactation of a wet nurse (a foster mother or caregiver) who is HIV negative shall be encouraged and such wet nurse shall be encouraged to remain HIV negative throughout the period of breastfeeding [12]. iv*)* Willingness to donate or receive donated milk (categorized as yes or no). v*)* Requirement of monetary remuneration for donating breast milk (categorized as yes or no). vi*)* Requirement of spousal consent before receiving or donating breast milk (categorized as yes or no). vii*)* Milk preference in serious medical conditions when own breast milk is contraindicated or impossible (categorized as donor breast milk and infant formulas). viii*)* Recommendation of donor breast milk to babies of other mothers (categorized as yes or no). See Table 1.

**Table 1:** Summary of definitions and measure used in the study

Variables	Measures
Dimensions measuring respondent’s sociodemographic characteristics	
Age	< 20; 20–29; 30–39; ≥ 40 years
Educational level	≤ 6 years; 6–12; > 12 years
Occupation	unemployed; unskilled; semi-skilled; skilled
Socioeconomic class	Low; middle; high
Religion	Christianity; Islam; traditional; others
Tribe	Igbo; Hausa; Yoruba; others
Marital status	Married; single
Spouse age	< 30 years; 30–39 years; 40–49; ≥ 50 years
Spouse education	≤ 6 years; 6–12; > 12 years
Dimensions measuring knowledge, awareness and attitude towards the concept of donor milk	
Ever heard of DBM^a^	Yes; no
Knowledge of the concept of DBM	None; partial; adequate
Awareness of policy on DBM	Yes; no
Willingness to donate/receive DBM	Yes; no
Requirement monetary remuneration	Yes; no
Requirement of spousal consent	Yes; no
Milk preference in medical condition	Donor breast milk; infant formulas
Will recommendation DBM	Yes; no

^a^Donor Breast Milk

### Data collection and analysis

Data collection was done using questionnaires administered by self and/or trained research assistants. Information were inputted into the relevant sections of the questionnaire and subsequently transferred into a Microsoft Excel Sheet. Distribution of the sociodemographic characteristics of respondents (predictor variables) and parameters related to perception of the concept of donor milk (outcome variable) were analyzed and reported in percentages. The Chi-square analysis was used to assess initial associations between the predictor and outcome variables. Binary logistic regression analysis was used to determine sociodemographic characteristics of respondents that predicted willingness to donate breast milk or receive donated breast milk among respondents. Measures of this association was presented as odd ratios (OR) and 95% confidence intervals (95% CI). Data analysis was done using IBM® SPSS version 21 (SPSS Inc., Chicago, IL) and statistical significance was set at *p* < 0.05. Respondents with grossly missing information were excluded from the data analysis.

## Results

### Description of study participants

**Fig. 1:**
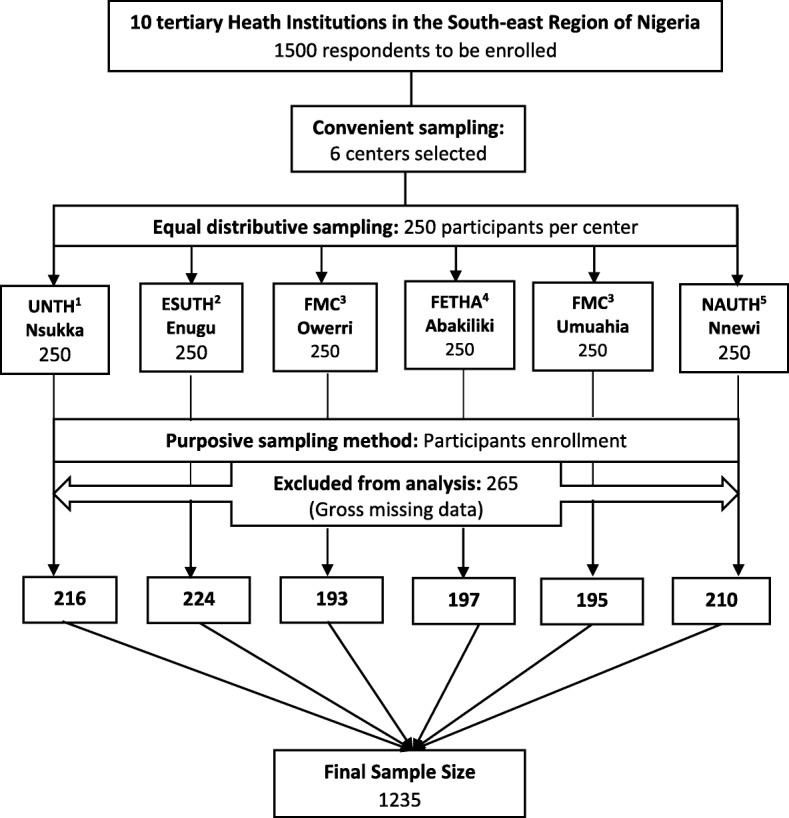
Summary of selection process for study participants. ^1^ UNTH- University of Nigeria Teaching Hospital, ^2^ ESUTH- Enugu State University Teaching Hospital, ^3^ FMC- Federal Medical Center; ^4^ FETHA- Federal Teaching Hospital Abakiliki, ^5^ NAUTH- Nnamdi Azikiwe University Teaching Hospital (©DIC Osuorah)

A total of one thousand five hundred (1500) questionnaires were administered through self and/or interviewer-based administration depending on the respondents’ abilities. Of these, two hundred and sixty-five respondents had grossly missing information and were excluded from the data analysis. One thousand two hundred and thirty-five women (1235) were successfully enrolled giving a response rate of 82.3% (Figure 1). Table 2 shows the characteristics of respondents. Approximately 40% were each within the 20–29 and 30–39-years age bracket. Another six and 11% were less than 20 and ≥ 40 years respectively. The mean age of the respondents in this study was 31.1 ± 7.3 and 38.9 ± 7.6 for their spouses. The majority (63%) of the mothers had a post-secondary school education and 68% were within the high socio-economic class. About a quarter of the study participants were unemployed and the remaining three quarters were employed in an unskilled (23%), semi-skilled (35%) and skilled (17%) occupational category. Almost all respondents (98%) were Christians and from the Igbo tribe (94%). Ninety-five percent were in a marital relationship and the other 5% were single i.e. never married, divorced or widowed.

**Table 2:** Characteristics of study respondents enrolled in the study

Characteristics	Variables	Number	Percentage
		(*n*)	(%)
Respondents age *(n = 1235)*	< 20 years	68	6
	20–29 years	509	41
	30–39 years	519	42
	≥ 40 years	139	11
Respondents education *(n = 1134)*	Primary education or less	60	5
	Completed secondary	362	32
	Post-secondary	712	63
Respondents occupation *(n = 1157)*	Unemployed	294	25
	Unskilled or low earners	264	23
	Semi-skilled or middle earners	403	35
	Skilled or high earners	196	17
Socio-economic class *(n = 1040)*	Low	120	12
	Middle	205	20
	High	715	68
Respondents religion *(n = 1194)*	Christianity	1173	98
	Islam	15	1.5
	Traditional	4	0.3
	Others	2	0.1
Respondents tribe *(n = 1170)*	Igbo	1099	94
	Hausa	14	1
	Yoruba	30	3
	Others	27	3
Marital status *(n = 1162)*	Married	1107	95
	Single	55	5
Spouses age *(n = 1040)*	< 30 years	290	24
	30–39 years	507	41
	40–49 years	358	29
	≥ 50 years	80	6
Spousal education *(n = 1062)*	Completed primary or less	87	8
	Completed secondary	363	34
	Post-secondary	612	58

### Perception of the concept of breast milk donation among respondents

Most of the respondents (61%) had never heard of the concept of breast milk donation. Of the 480 (39%) that had heard of it, only 81/480 (17%) were aware of government policies on breast milk donation. Source of information for respondents that have heard of donor milk were doctors 176/679 (25%), nurses and other health workers 145/679 (21%), electronic and print media 110/679 (16%) and internet users 88/679 (13%). Others include books 69/679 (10%), religious houses 38/679 (6%) and from family members 67/679 (9%). See Table 3. When asked to define the concept of donor breast milk/feeding, 186/759 (25%) had no knowledge of what it means, 494/759 (65%) had partial knowledge, while only 79/759 (10%) had a correct idea of the concept of donor milk donation. Some of the partially correct explanation given by respondents include but not limited to:

[AB 45 years]: *“To be fed by a different mother that is not yours”-;* [CD 36 years]: *“A case were the mother is sick or dead and another woman breast feeds her child”-;* [EF 21 years]: *“Breastfeeding a baby who needs it-;* [GH 67 years]*"A situation where your sister or cousin breastfeeds your child”-;*

**Table 3:** Parameters on knowledge, awareness and attitude towards the concept of donor breast milk amongst respondents

Parameters of Donor Breast milk	Variables	Number	Percentage
		(*n*)	(%)
Ever heard of breast milk donation *(n = 1225)*	Yes	480	39
	No	745	61
Knowledge of the concept of donor milk *(n = 759)*	None	186	25
	Partial	494	65
	Adequate	79	10
Awareness of policy on milk donation *(n = 1179)*	Yes	81	7
	No	1098	93
Willingness to receive donated milk (*n* = 1172)	Yes	707	60
	No	465	40
Would require monetary remuneration for donating breast milk *(n = 1134)*	Yes	150	13
	No	984	87
Would require spousal consent before reeving or donating breast milk *(n = 1134)*	Yes	885	80
	No	220	20
Preference for milk donor *(n = 948)*	Relatives	853	90
	Non-relatives	95	10
Would recommend donor breast milk for babies of other mothers *(n = 1176)*	Yes	587	50
	No	589	50
Milk preference in serious medical conditions where breastfeeding is contraindicated or impossible *(n = 1040)*	Donor Breast milk	555	55
	Infant formula	463	45
Experiences excessive breast milk flow during lactation *(n = 1139)*	Yes	514	55
	No	625	45

Some of the correct definitions included but also not limited to:

[BC 55 years]: *“Screened mother breast milk to feed another woman’s baby”-;* [XY 27 years]: *“Breast milk donated to babies not yours”-;* [FT 34 years]: *“Expressing or extracting breast milk and donating it”-; and* [SB 45 years]: *“Mothers donating breast milk for other mothers who do not wish to feed their babies with their own breast milk”-;*

Five hundred and fourteen study participants (45%) said they usually experience excessive breast milk flow during lactation. Seven hundred and seven (60%) respondents expressed willingness to receive donated breast milk if the need arises while the remaining 465 (40%) would prefer not to receive breast milk. Among the latter group, 195/465 (43%) would receive donated breast milk if the donor is well screened and deemed safe. A hundred and fifty of them (13%) will desire financial remuneration to donate while 885 (80%) will require spousal consent to either donate or receive breast milk in case of need. A significant proportion of those who require spousal consent were more educated (postsecondary 84% vs. secondary 76% vs. primary 67%, *p* = 0.001), of higher socio-economic class (high 83% vs. middle 76% and low 71%, *p* = 0.003) and in the lower occupational category (unemployed 87% vs. unskilled 72% vs. semi-skilled 81% and skilled 82%, *p* = 0.001).

Almost all respondents 1199 (98%) believed breast milk was the best feed for the newborns. The most common reasons for this belief was that breast milk was nutritionally superior to infant formula 829/3669 (23%) and protective against disease 797/3669 (22%). Others included affordability (14%), convenience (12%), warmth (10%), bonding (12%) and other unspecified reasons (7%). Based on these reasons, 587 (50%) of the respondents supposed they would recommend donated breast milk to other mothers who may need it. Five hundred and fifty-five respondents (55%) would prefer donated breast milk to infant formula if there is a medical condition that prevents the use of their own milk and about 9 in 10 of the respondents would prefer the donation of breast milk to be from a relative. See Table 3.

Finally, respondents who expressed unwillingness to donate breast milk or use donor milk were asked to give reasons for their perceived belief for and against the use of donor milk. Table 4 shows summarizes some of the reason given by the respondents.

**Table 4:** Perceived reasons for and against the belief and use of donor milk amongst respondents

Reasons for Donor Milk^a^	*n* (%)
• Mothers breast milk is not flowing	127 (31.4)
• Baby is crying, and mother is not around	75 (18.6)
• Mother is sick and cannot breast feed	139 (34.4)
• Mothers breast milk is not safe for baby	12 (3.0)
• Baby is not gaining weight because of inadequate milk	4 (1.0)
• Donor milk is convenient and safe	1 (0.2)
• To prevent breast from sagging	1 (0.2)
• Other unspecified reasons	45 (11)
Total	404
Reasons against Donor Milk^a^	*N* (%)
• Donor milk is not culturally accepted	191 (11.8)
• Donor milk is not religiously accepted	109 (6.7)
• There is risk of infection transmission	426 (26.3)
• Not sure its hygienically prepared	217 (13.4)
• Concerns about HIV, Hepatitis B and C	241 (14.9)
• Social and societal stigma	92 (5.7)
• Not a common practice and generally unacceptable	160 (9.9)
• Fear of transmission of bad genetic traits	185 (11.3)
Total	**1621**

^a^Multiple responses were allowed

### Maternal sociodemographic factors and the concept of breast milk donation

Of the maternal sociodemographic characteristics considered in the study, age of respondents, religion, tribe of origin and marital status were significantly associated with being aware of the policy on breast milk donation. Mothers that were older (≥ 40 yrs. 13%, 30–39 yrs. 7%, 20–29 yrs. 5% and <  20 yrs. 7%; *p* = 0.008), non-Christians (Christian 6%, vs. other religion 22%; *p* = 0.001), non-Igbos (Igbos 6%, Hausas 21%, Yorubas 20%, others 19%; *p* = 0.001) and those unmarried (28% vs. 6%; *p* = 0.001) were more aware of policies related to breast milk donation.

Furthermore, only respondent’s age and occupational status was significantly associated with adequate knowledge of donor milk (Table 3). More respondents below the age of 20 years (19%) had adequate knowledge of the concept compared to those in other age categories (≥ 40 yrs. 8%, 30–39 yrs. 9%, and 20–29 yrs. 12%; *p* = 0.049), educational attainment (post-secondary 42%, secondary 31% and primary 45%; *p* = 0.001), occupational status (unemployed 31%, unskilled 31%, semi-skilled 48% and skilled 44%; *p* = 0.0010).

Finally, more respondents with lower educational attainment, primary (27%) would require financial inducement to donate breast milk compared to those with secondary (15%) and postsecondary (11%) education; *p* = 0.002. Similarly, non-Christians (26%) were 2.4 times more likely to require financial inducement before donating breast milk than Christian (13%); *p* = 0.004]. See Table 5.

**Table 5:** Sociodemographic factors and association with parameters accessing acceptability of the concept of donor milk

Respondent Variables	Willingness to donate or receive donated Breast milk				Awareness of government policy on Donor milk				Financial remuneration for donation of milk				Knowledge of Donor Milk				
	Yes	No	*n*	*p*	Yes	No	n	*p*	Yes	No	*n*	*p*	None	Partial	adequate	*n*	*p*
Age (years)	707	465	1172		81	1098	1179		150	984	1134		186	494	79	759	
< 20	32(53)	29(47)	61	.121	4(7)	57(93)	61	.008	9(15)	50(85)	59	.851	9(26)	20(57)	6(17)	35	.049
20–29	287(52)	198(48)	485		24(5)	465(95)	489		65(14)	403(86)	468		85(28)	187(61)	37(12)	309	
30–39	314(64)	178(36)	492		35(7)	459(93)	494		61(13)	417(87)	478		79(25)	215(67)	29(9)	323	
≥ 40	74(55)	60(45)	134		18(13)	117(87)	135		15(12)	114(88)	129		13(14)	72(78)	7(8)	92	
Education	660	420	1080		73	1014	1087		138	904	1042		184	454	78	716	
Primary	31(53)	27(47)	58	.000	7(12)	52(88)	59	.051	15(27)	40(73)	55	.002	5(15)	27(82)	1(3)	33	.098
Secondary	181(53)	160(47)	341		15(4)	326(96)	341		49(15)	279(85)	328		57(30)	111(59)	19(10)	187	
Postsecondary	448(66)	233(34)	681		51(7)	636(93)	687		74(11)	585(89)	659		122(25)	316(64)	5811)	496	
Occupation	673	428	1101		78	1029	1107		139	69	1063		183	466	76	723	
Unemployed	173(62)	107(38)	280	.004	11(4)	268(93)	279	.076	32(12)	16(36)	269	.192	67(37)	92(51)	21(12)	180	.000
Unskilled	130(54)	113(46)	243		16(7)	228(93)	244		38(16)	13(32)	237		23(18)	93(71)	15(11)	131	
Semi-skilled	246(63)	142(37)	388		33(8)	358(92)	391		41(11)	40(24)	377		55(20)	190(69)	3111)	276	
Skilled	124(65)	66(35)	190		18(9)	175(91)	193		28(16)	154(84)	180		38(28)	89(65)	9(7)	136	
SES	616	374	990		69	927	996		131	824	955		177	404	68	649	
Low	60(52)	56(48)	116	.019	10(9)	109(91)	117	.141	20(19)	87(81)	107	.134	17(29)	36(61)	6(10)	59	.980
Middle	110(60)	75(40)	185		7(4)	182(96)	189		29(16)	154(84)	182		30(25)	77(65)	12(10)	119	
High	446(65)	243(35)	689		52(8)	638(92)	690		82(12)	583(88)	665		130(28)	291(62)	50(10)	471	
Religion	707	465	1172		81	1098	1179		150	984	1134		186	494	79	759	
Christianity	677(61)	436(39)	1113	.127	68(6)	1052(94)	1120	.000	135(13)	941(87)	1076	.004	184(25)	475(65)	72(10)	731	.080
Others	30(51)	29(49)	59		13(22)	46(78)	59		15(26)	43(74)	58		2(7)	19(68)	7(25)	28	
Tribe	671	439	1039		73	1044	1117		141	932	1073		186	465	74	725	
Igbo	628(52)	411(40)	1039	.763	59(6)	988(94)	1047	.000	130(13)	875(87)	1005	.265	174(26)	436(64)	69(10)	679	.940
Hausa	9(64)	5(36)	14		3(21)	11(79)	14		4(29)	10(71)	14		2(25)	5(63)	1(12)	8	
Yoruba	16(53)	14(47)	30		6(20)	24(80)	30		5(17)	24(83)	29		4(19)	14(67)	3(14)	21	
Others	18(67)	9(33)	27		5(19)	21(81)	26		2(8)	23(92)	25		61(35)	10(59)	1(6)	17	
Marital status	667	431	1048		74	1035	1109		138	927	1065		185	459	74	718	
Married	636(61)	412(39)	1048	.631	59(6)	996(94)	1055	.000	130(13)	881(87)	1011	.677	180(27)	429(63)	68(10)	677	.106
Single	31(57)	23(43)	54		15(28)	39(72)	54		8(15)	46(85)	54		5(12)	30(73)	6(15)	41	

### Determinants of willingness to donate breast milk or accept donated breast milk

Tables 5 and 6 show cross-tabulation of maternal factors associated with willingness to donate breast milk or accept donated milk. Maternal education (*p* = 0.001), occupational strata (*p* = 0.004), knowledge of the concept of donor milk (*p* = 0.001), awareness of government policies (*p* = 0.001), seeking financial remuneration (*p* = 0.001) and spousal consent to donate/receive donated milk (*p* = 0.001) were significantly associated with willingness of respondents to donate and/or receive donated milk. Others include preference for donor (*p* = 0.001), donor tested and safe (*p* = 0.001), and milk preference in serious medical condition (*p* = 0.001).

**Table 6:** Other associated parameters amongst respondents that determines willingness to donate breast milk and/or receive donated breast milk

Parameters	Willingness to donate/receive DBM			
	Yes *n* (%)	No *n* (%)	Total	*p*
Knowledge of donor breast milk	*n* = 480	*n* = 258	*n* = 738	
None	144 (78)	40 (22)	184	0.001†
Partial	295 (62)	183 (38)	478	
Adequate	41 (54)	35 (46)	76	
2				
Awareness of Policies regarding DBM	*n* = 704	*n* = 460	*n* = 1164	
Yes	66 (82)	15 (18)	81	0.001†
No	638 (59)	445 (41)	1083	
Would require financial numeration to donate	*n* = 702	*n* = 418	*n* = 1120	
Yes	126 (84)	24 (16)	150	0.001†
No	576 (59)	394 (41)	970	
Have heard of Donor breast milk	*n* = 701	*n* = 464	*n* = 1165	
Yes	283 (61)	178 (39)	461	0.492
No	418 (59)	286 (41)	704	
Spouse consent to donate/receive DBM	*n* = 694	*n* = 400	*n* = 1094	
Yes	613 (70)	265 (30)	878	0.001†
No	81 (38)	135 (62)	216	
Experience excessive milk flow	*n* = 681	*n* = 448	*n* = 1129	
Yes	320 (62)	193 (38)	513	0.197
No	361 (59)	255 (41)	616	
Preference for donor	*n* = 629	*n* = 306	*n* = 935	
Relatives	587 (70)	258 (30)	845	0.001†
Non-relatives	42 (47)	48 (62)	90	
Would accept DBM if donor tested and safe	*n* = 579	*n* = 441	*n* = 1020	
Yes	245 (42)	339 (58)	584	0.001†
No	334 (77)	102 (23)	436	
Milk preference in serious medical conditions	*n* = 571	*n* = 420	*n* = 921	
Donor milk	425 (77)	127 (23)	552	0.001†
Infant formula	146 (33)	293 (67)	439	

*DBM* donated breast milk, †*P* value is statistically significant

Table 7 shows the multivariable logistic regression analysis of maternal characteristics and their willingness to donate or receive donated breast milk. After adjusting for other maternal variables of interest, only having knowledge of the concept of donor milk (*p* = 0.001), seeking financial remuneration (*p* = 0.001) and milk preference in serious medical condition (*p* = 0.001) retained significance as determinants of willingness to donate or accept donated breast milk among respondents (Table 7). Respondents with adequate knowledge of the concept of donor milk were about 10.8 times OR 10.76, CI 2.78, 23.67; (*p* = 0.001) while those with partial knowledge were 3.41 times OR 3.41, CI 0.98, 11.84; (*p* = 0.054) more likely to donate breast milk or accept donated breast milk for their newborns. Similarly, respondents that indicated financial remuneration as perquisite for breast milk donation were 0.18 less likely to donate their breast milk compared to those that would donate without charge OR 0.18, CI 0.10, 0.44; (*p* = 0.001). In other words, respondents willing to donate without financial incentives were about 5.5 times more likely to donate their breast milk compared to those that require financial remuneration to donate. Lastly, it was noted that in serious medical conditions where respondents cannot breastfeed their babies or situations where their breast milk is contraindicated, those that prefer donor milk to infant formula were 6.64 times more willing to donate their breast milk or receive donor milk than those that prefer infant formula in such conditions (OR 6.64, CI 2.87, 15.36; *p* = 0.001).

**Table 7:** Regression analysis of factors significantly associated with willingnes*s* to donate breast milk or receive donated breast milk among respondents

Variables	Crude OR [95% CI]	*P*	Adjusted OR†^2^ [95% CI]	*P*
Mothers education				
Completed primary or less	3.89 (0.55, 12.22)	0.371	2.99 (0.33, 12.01)	0.330
Completed secondary	1.49 (0.63, 3.50)	0.365	1.16 (0.42, 3.16)	0.777
Post-secondary	1	–	1	–
Mothers occupation				
Unemployed	1	–	1	–
Unskilled or low earner	2.18 (0.86, 5.48)	0.099	2.41 (0.89, 6.49)	0.081
Semi-skilled or middle earner	1.69 (0.74, 3.87)	0.213	1.99 (0.81, 4.92)	0.132
Skilled or high earner	2.13 (0.85, 5.32)	0.105	2.62 (0.98, 7.03)	0.055
Mothers socioeconomic class				
Low	1	–	1	–
middle	0.20 (0.04, 1.06)	0.058	0.11 (0.01, 1.14)	0.064
High	1.41 (0.57, 3.49)	0.452	1.07 (0.31, 3.71)	0.917
Knowledge of donor milk				
None	1	–	1	–
Partial	3.94 (1.34, 11.56)	0.013†	3.41 (0.98, 11.84)	0.054
Adequate	12.22 (3.53, 24.31)	0.001†	10.76 (2.78, 23.67)	0.001†
Awareness of donor milk policies				
Yes	0.58 (0.22, 1.51)	0.259	0.47 (0.16, 1.36)	0.163
No	1	–	1	–
Financial remuneration involved				
Yes	0.20 (0.12, 0.47)	0.001†	0.18 (0.10, 0.44)	0.001†
No	1	–	1	–
Spouse consent to donate/receive				
Yes	1	–	1	–
No	2.04 (0.96, 4.33)	0.063	2.16 (0.99, 4.73)	0.052
Preference for donor				
Relatives	1	–	1	–
Non-relatives	1.93 (0.73, 5.13)	0.188	2.16 (0.78, 5.96)	0.136
Donor tested and safe				
Yes	1.11 (0.54, 2.29)	0.782	1.03 (0.49, 2.18)	0.931
No	1		1	–
Milk preference in medical conditions				
Donor milk	6.87 (3.07, 15.39)	0.001†	6.64 (2.87, 15.36)	0.001†
Infant formula	1	–	1	–

† *P* value is statistically significant, †^2^ Adjusted for maternal age, tribe, religion, marital status and spousal factors

## Discussion

This study reports a low knowledge of the concept donor breast milk and low awareness of policy regarding the use of Donor Breast Milk (DBM) in Nigeria but a relatively high willingness of mother to participate in the concept. This finding is similar to the results of a comparable survey on 198 mothers in a tertiary hospital in south-south Nigeria which showed that only 25.8% have heard about the concept of DBM but 59.1% strongly believed that human milk banking is necessary to assist orphaned and abandoned babies [13]. Another study that surveyed 448 mothers in Izmir, Turkey also showed that even though only 41.6% were aware of the concept of milk banking, 71.3% were willing to receive milk bank services and 68.8% were willing to donate their breastmilk [14]. Correspondingly, it was noted in our study that the most common source of information about donor breast milk was from healthcare workers which is identical to the 46.1% reported in another study in south-south Nigeria [13]. These is however contrarily to the finding of the study in Turkey where the media accounted for 85.7% of the information source of DBM among respondents. These differences may be related to the better availability of electronic media to mothers in Turkey where for instance 46% of women have access to internet and other electronic media compared to 28% in Nigeria [15, 16].

Like this study, the studies in Benin and Izmir showed a major concern about infection transmission was the common reason mothers were unwilling to accept donated breast milk for their babies [13, 14]. The authors of these studies specifically reported that 59.1% and 62.2% of surveyed mothers in Benin and Izmir respectively, were unwilling to receive donated breast milk stating the risk of contracting infections as a major concern. As noted in our study, the number of mothers who expressed unwillingness to use donor breast milk significantly reduced when they were assured that donor will be screened and tested for transmittable infections. This further strengthens the need for proper enlightenment of mothers and their families about the processes involved in the concept of human breast milk banking. In the same vein, our study showed that less than a tenth of respondents mentioned religion as a concern for their unwillingness to donate or receive donated breast milk, unlike in the Turkey study where a sizable proportion of mothers surveyed expressed religion as a barrier to acceptability of the concept of DBM [14]. To reduce or completely eradicate the distracting influence of religion on the well documented benefit of breast milk, relevant government authorities needs to involve faith and religious leaders in the campaign for DBM in order to eliminate religious prejudices and increase the acceptability of the concept.

Other reasons for unwillingness to receive donor breast milk documented in our study includes cultural unacceptability, unhygienic preparation, societal stigma, generally unacceptability, fear of transmission of bad genetic traits etc. These are realistic and honest concerns that can be allayed through targeted enlightenment programs and sharing of real-life success stories.

Due to the setting where this study was conducted, it is worth mentioning that the willingness to donate or receive donor breast milk was significantly associated with spousal consent even though the association lost significance on adjusted logistic regression analysis. Several studies in Nigeria and other developing countries have reported that most women seek permission from their spouse for use of health-related facilities [17–19]. The need for consent by women from their spouses for health-related matters in most developing settings is an unfortunate but unsurprising fact in a patriarch society like Nigeria where polygamy is common and women are usually dependent on their spouse for most decisions due to inequalities in income and age. To the best of authors knowledge, no study has specifically investigated the association between willingness to donate/receive donor breast milk and spouse consent in a developing setting.

We found that knowledge of the donor breast milk concept, not being interested in financial compensation for donating breast milk and preference of donor breast milk over infant formulas were predictive of the mothers that would be potential breast milk donors. This is in line with the findings from a similar multi-center study in eastern Ethiopia which showed that acceptance of donor milk banking was more likely among mothers who had heard about donor milk banking and wet-nurses [20]. Another study in Rio de Janeiro, Brazil found that enhancing the understanding of the donor breast milk concept through encouragement by healthcare professionals, relatives, or friends, receiving information on breast milk expression by the primary health care unit, and receiving help from the unit professionals to breastfeed were associated with a higher prevalence of breast milk donation [21]. These findings are hardly surprising as conceptual understanding of a program or concept causes acceptance and participation where appropriate.

Furthermore, our study noted that mothers without monetary motives were more willing to donate their breast milk compared to those that require financial remuneration. Fortunately, only a small proportion of mothers in our survey indicated interest in monetary compensation before donating their breast milk. This is particularly not unexpected in a developing country like Nigeria where people donate their blood for financial gains [22] and where, over 65% women live in extreme poverty [23]. Financial remuneration for breast milk donation has been the subject of continuing research which has produced some conflicting results. Nonetheless, the Human Milk Banking Association of North America (HMBANA) endorses non- profit donor milk banking to ensure that a valuable healthcare resource is allocated in an ethical and safe manner, keeping the safety and needs of the recipient and donor paramount [24]. In addition, HMBANA has identified several problems associated with introducing the profit motive to milk banks. These includes adulterating milk to increase volume and placing the infant of the lactating mother at risk if she feels pressure to provide a certain volume of milk to a bank or a recipient rather than feeding her own infant [19]. Bloom noted in a study that adulteration of milk for financial gain led to rejection of 73 out of 4935 breast milk donations and that the dilution rejection rate was significantly higher in donors that required financial remuneration [25]. Also, Keim et al. reported that in 102 purchased samples advertised online as breast milk, eleven contained bovine DNA and ten of these had a level of bovine DNA consistent with human milk mixed with at least 10% cow’s milk [26].

Finally, we reported that mothers who would prefer donor breast milk over infant formula for their newborns in situations where they cannot breast their own babies were more likely to donate their breast milk compared to those that favored infant formulas. It is reasonable to assume that mothers that prefer donor breast milk over infant formula would most probably have reasonable knowledge of the social, economic and health benefits of breast milk and would naturally be more willing to donate their own breast milk for mothers and infants in need. The World Health Organization recommends donor breast milk as the next preferred option in situations where women are not able to provide their infants with enough amounts of their own breast milk [27]. Unfortunately, developing countries lag the rest of the world in establishing and promoting human milk banks. As a matter of fact, there is currently no functional human milk bank in the whole of the West African sub-region [28].

### Limitations

Firstly, the cross-sectional design of this study makes it difficult to establish causal temporal relationships between the predictor (sociodemographic parameters of mothers) and outcome (willingness to donate or receive donor breast milk) variables considered in this study. Secondly, because only mothers were enrolled in our study, the views of health professionals, policy makers, community leaders and other stakeholders in the breast milk banking service were left out thus eliminating an important source of data regarding this concept. Finally, some inaccurate information from respondents might have resulted in misclassification of variables and subsequent misinterpretation of study conclusions. The authors therefore recommend that the findings of this study be interpreted in the light of these limitations.

## Conclusions

In conclusion, our study found that the knowledge of the concept of donor breast milk and awareness of policies recommending its practice in Nigeria is low, but the prospect of its acceptability is high among mothers surveyed in south-east Nigeria. Targeted public enlightenment and education to mothers and their families by relevant government agencies in collaboration with clinicians, community and religious leaders about the concept of donor breast milk may help increase the acceptance and practice of donating breast milk and/or use of donated breast milk among mothers in the region.

## Abbreviations

CI: Confidence Interval
DBM: Donor Breast Milk
HIV: Human Immunodeficiency Virus
HMB: Human Milk Bank
HMBANA: Human Milk Banking Association of North America
NICE: National Institute of Health and Clinical Excellence
OR: Odd ratio
UN: United Nations
UNICEF: United Nation Children’s Fund
WHO: World Health Organization
